# Supplementary material for: hiPSC-Derived Schwann Cells Influence Myogenic Differentiation in Neuromuscular Cocultures
Source: Cells. 2021 Nov 24;10(12):3292. doi: 10.3390/cells10123292 (PMC8699767; doi:10.3390/cells10123292)
Supplement: Supplementary file 1 [file cells-10-03292-s001.zip › cells-1453061-supplementary.pdf]

Supplementary Data

hiPSC-derived Schwann cells influence myogenic differentiation in neuromuscular cocultures

Sarah Janice Hörner, Nathalie Couturier, Roman Bruch, Philipp Koch, Mathias Hafner, Rüdiger Rudolf

Supplementary Figure S1. Representative micrographs of marker protein immunostainings for hiPSC-derived motoneurons p.2

Supplementary Figure S2. Schwann cell differentiation additional tests……………………………………..p.3

Supplementary Figure S3. Representative micrographs of marker protein immunostaining panel for different Schwann cell maturation media conditions……………………………………………………..…p.4

Supplementary Figure S4. Cell types colocalize and align in tricultures…………………………………..…p.5

Supplementary Figure S5. Completely hiPSC-derived NMJ tricultures…………………………………………p.6

Supplementary Figure S6. Cocultures increase percentage of AChR clusters positive for rapsyn staining……p.7

Supplementary Table S1. Primary antibodies used for immunofluorescence stainings……..………………..…p.8

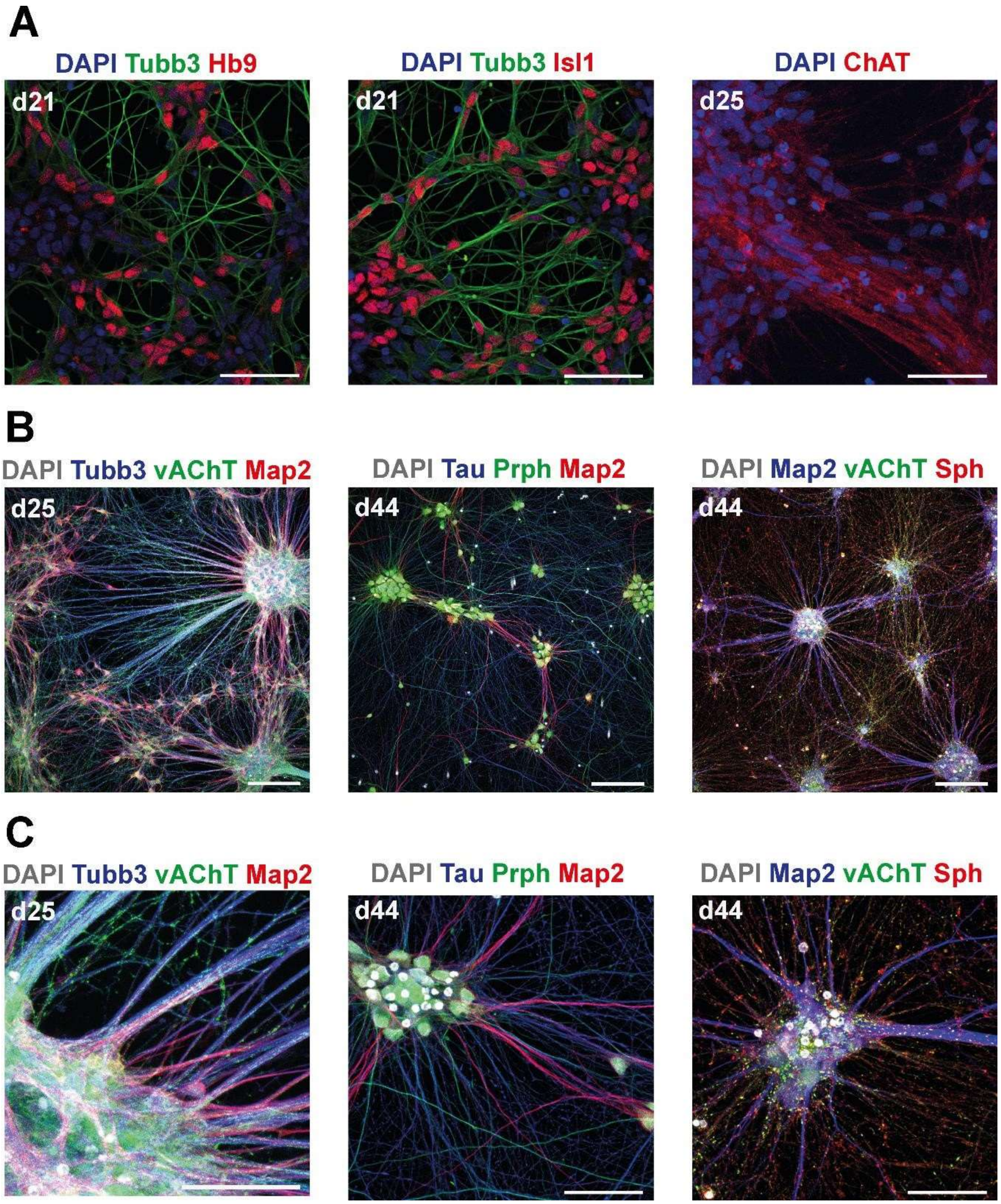

Supplementary Figure S1. hiPSC-derived MN express major neuronal and motoneuronal markers. hiPSC were differentiated according to the protocol shown in Fig. 2A, fixed at time points as indicated on the top left of panels and then immunostained for markers βIII-tubulin (Tubb3), Hb9, Isl1, choline acetyltransferase (ChAT), vesicular acetylcholine transporter (vAChT), Map2, tau, peripherin (Prph), or synaptophysin (Sph); as displayed on top of panels. Nuclei were labeled with DAPI and shown in blue (A) or white (B-C). Scale bars: 50 µm (**A** and **C**); 100 µm (**B**).

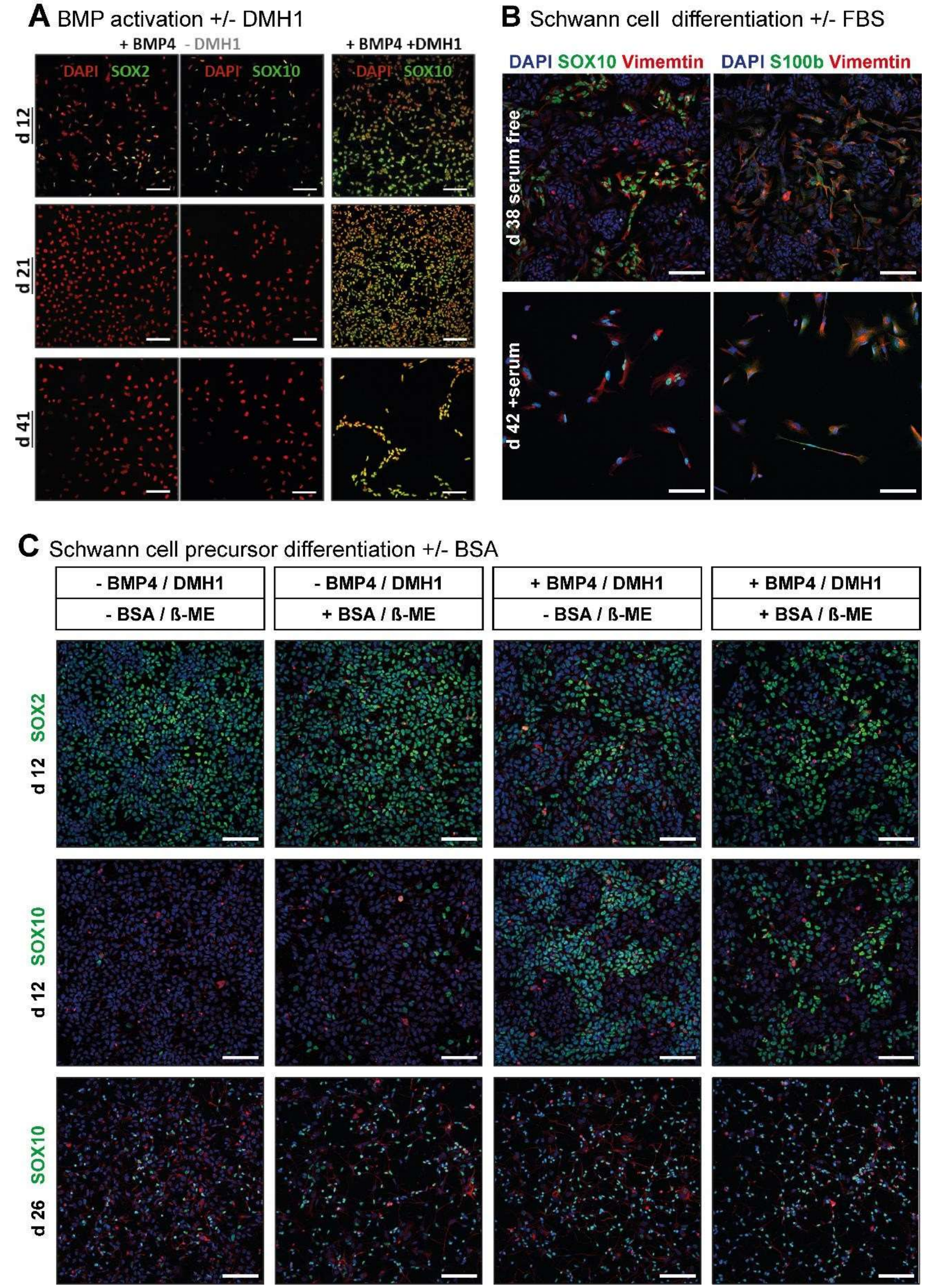

Supplementary Figure S2. Schwann cell differentiation additional tests. (**A**) Comparison of cells differentiated with or without DMH1 addition during BMP4 treatment on days 6 – 12, stained for SOX2 or SOX10 at timepoints as indicated. Scale bars: 100 µm. (**B**) Schwann cells were differentiated in medium containing 1 % FBS from day 24 on or alternatively in serum-free medium containing 1× N2 and 1× SM1, and stained for Schwann cell markers SOX10 and S100b. Quantification via image segmentation analysis showed 18.7 ± 5.7 % of cells stained positive for SOX10, and 20.6 ± 6.3 % positive for S100b at day 38 in serum free medium. Scale bars: 100 µm. (**C**) Comparison of Schwann cell precursor differentiations with and without 0.005 % BSA and 0.11 mM β-mercaptoethanol for days 0 – 24; both conditions with and without BMP tuning from days 6 - 12. Representative micrographs show DAPI (blue), Vimentin (red) and SOX2 or SOX10 (green) as indicated. Scale bars: 100 µm.

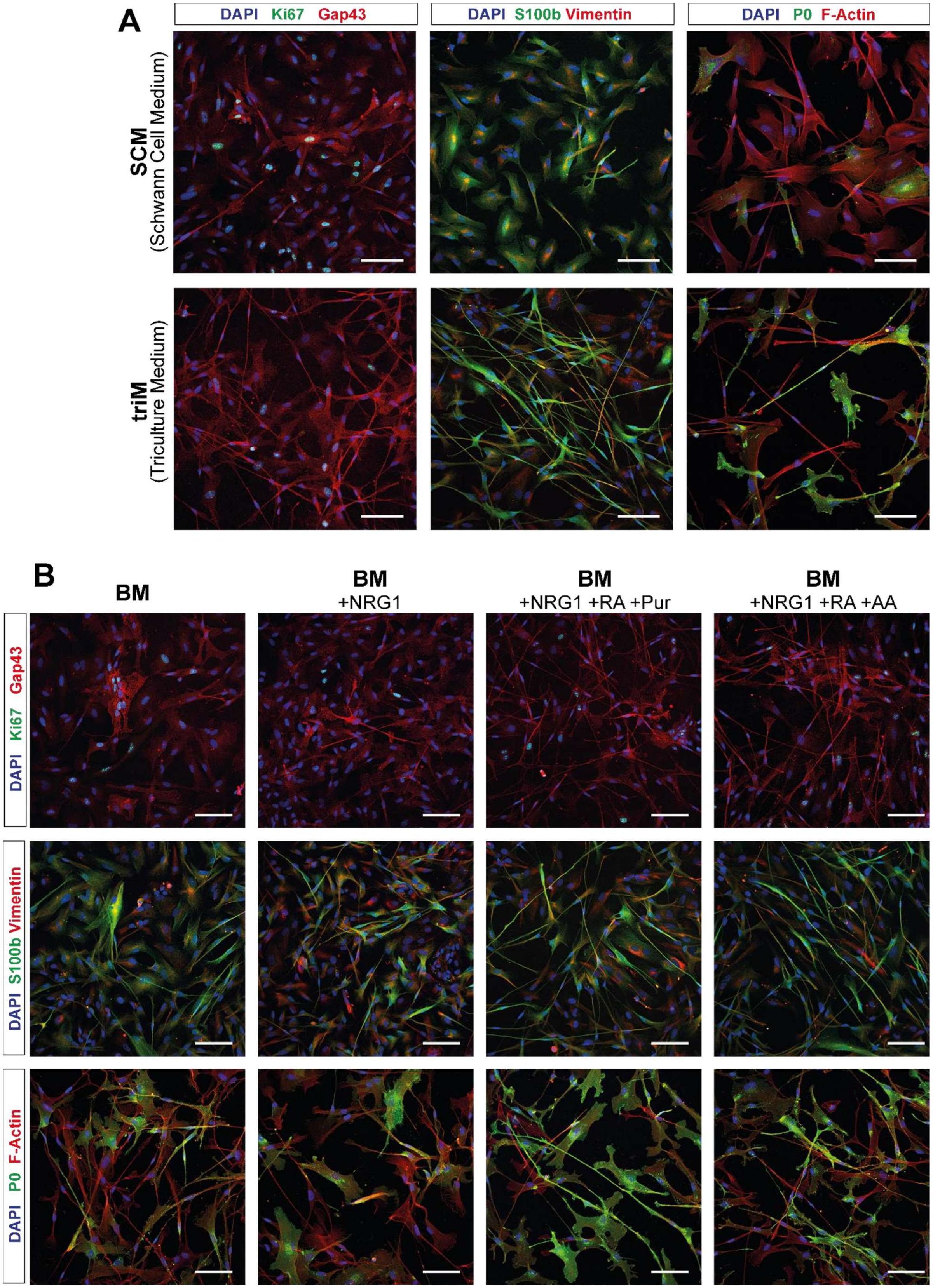

Supplementary Figure S3. Representative micrographs of marker protein immunostaining panel for different Schwann cell maturation media conditions. (**A**) Comparison of cells cultured in Schwann cell medium (SCM) or in fully supplemented triculture medium (triM) for 3 days. (**B**) Cells cultivated for 3 days in triculture basal medium (BM) or BM with additional factors as indicated. Stainings indicated in panels. Scale bars: 100 µm.

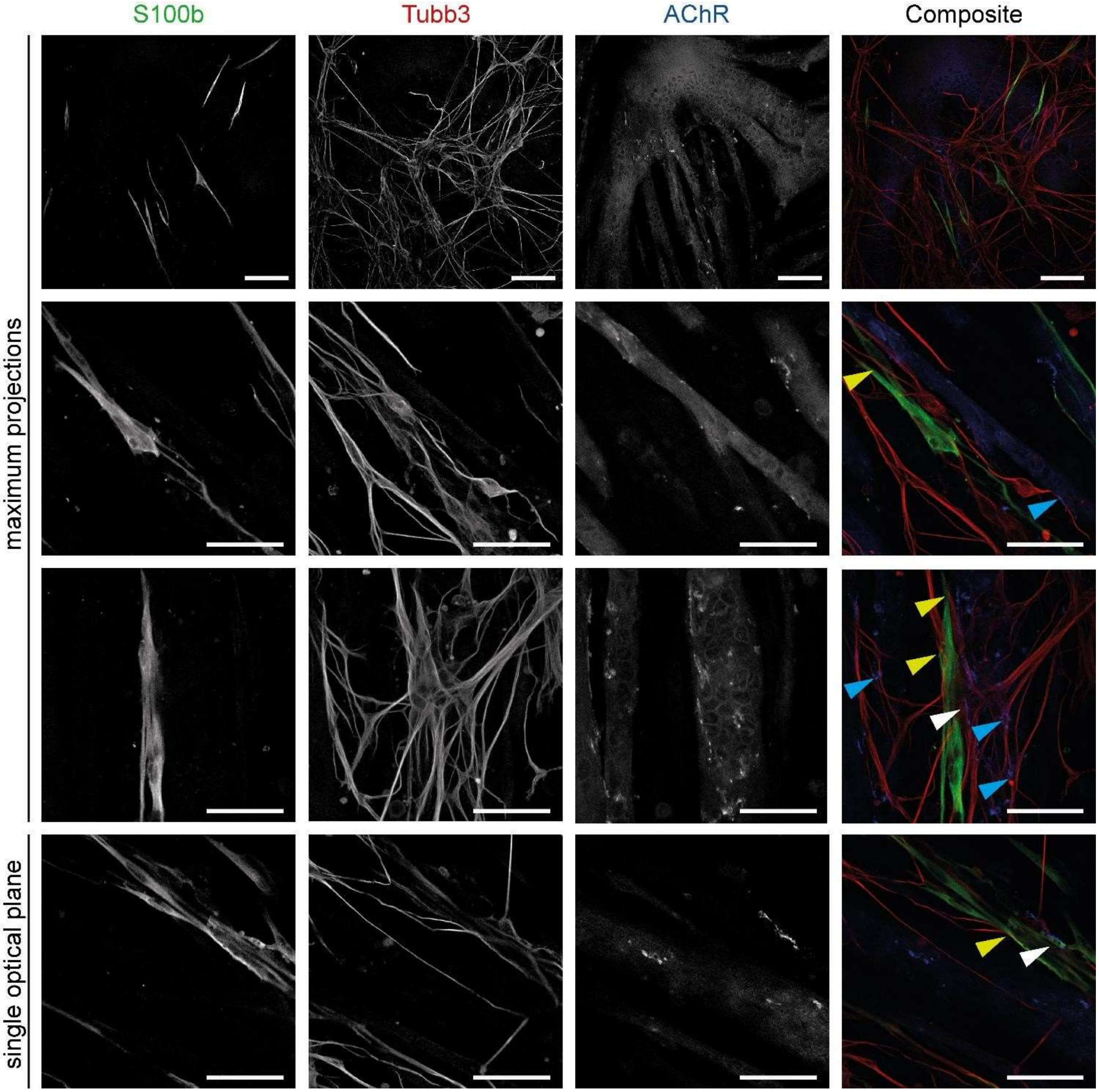

Supplementary Figure S4. Cell types colocalize and align in tricultures. Representative confocal micrographs out of several independent triculture experiments (n = 5) with Schwann cells shown in green (S100b), motoneurons in red (Tubb3), and αBTX-labelled AChR shown in blue in composite images. Yellow arrows: examples of Schwann cells aligned with motoneurons; cyan arrows: colocalization of motoneurons with AChR; white arrows: colocalization of both motoneurons and Schwann cells with AChR. Last row demonstrates colocalizing cells in a single confocal plane. Scale bars: first row 100 µm; others 50 µm.

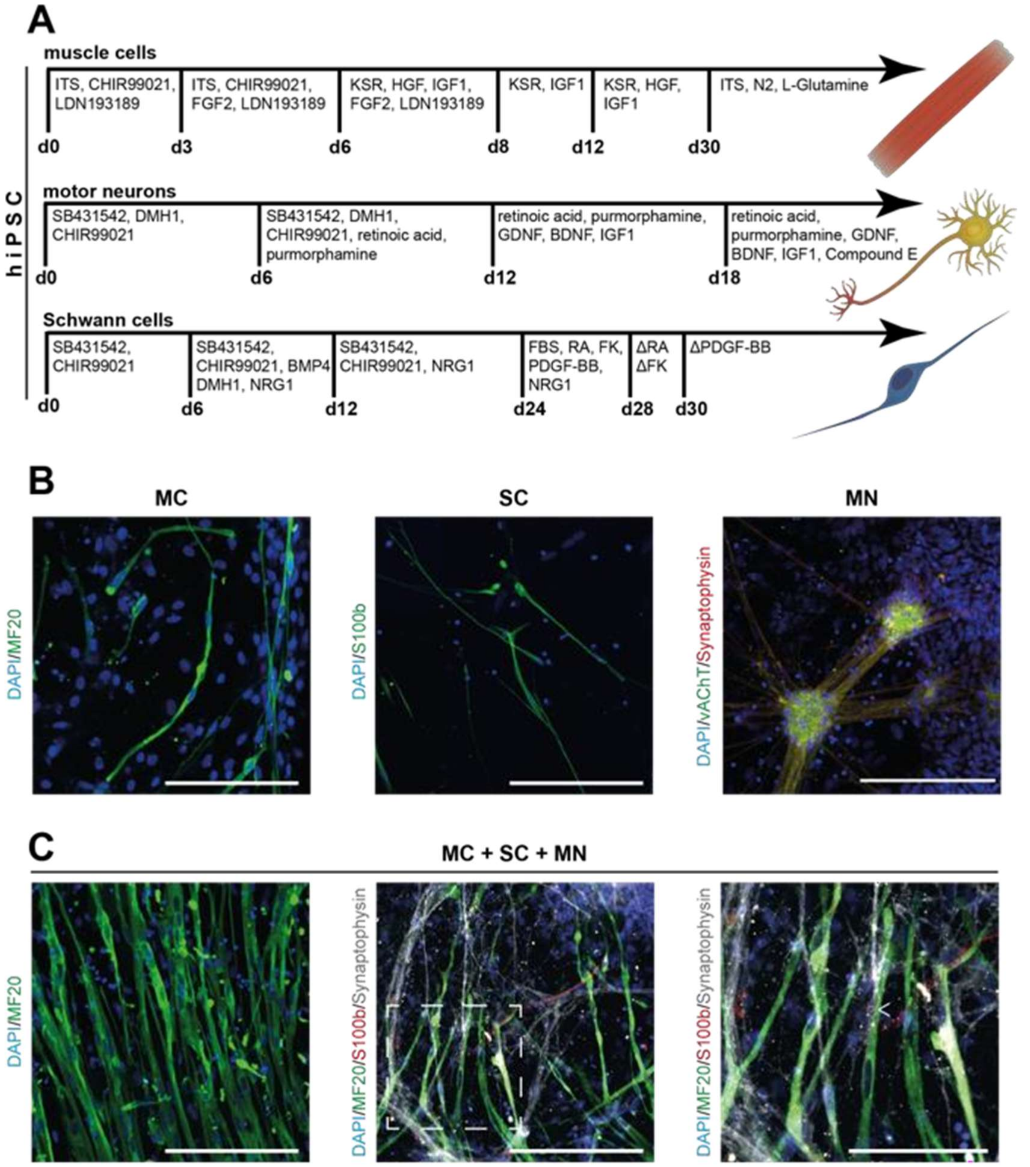

Supplementary Figure S5. Completely hiPSC-derived NMJ tricultures. (**A**) Additionally to motoneurons (MN) and Schwann cells (SC), skeletal muscle cells were differentiated from hiPSC. hiPSC-derived myogenic progenitors were generated according to Chal et al., 2016 [37]. After 30 days of primary differentiation, myogenic progenitors were harvested and seeded onto Matrigel (heSC-qualified, 1:100 in DMEM/F12) coated 4-well Ibidi® slides. Myogenic progenitors were maintained 4 days in proliferation medium containing SkBMTM-2, 10 % FBS, 40 ng/mL Dexamethasone, 2 mM L-Glutamine, 3 ng/mL human EGF, 15 ng/mL Amphotericin-B, 30 µg/mL Gentamycin. Then, a concentrated layer of Matrigel was added and progenitors were kept in proliferation medium for 15 additional days. Terminal differentiation was induced using N2-based medium as described in Chal et al. 2016 and medium was refreshed every other day. After 10 days, hiPSC-derived MN and SC were added and cultures switched to triculture medium as described. Medium was refreshed partially every other day and cultures were fixed after 10 more days. (**B**) Immunofluorescence staining of monocultures of hiPSC-derived muscle cells (MC), MN, and SC, stained for marker proteins as indicated. Scale bars: 200 µm. (**C**) Immunofluorescence staining of tricultures after 10 days involving hiPSC-derived MC, SC, and MN. Nuclei are represented in blue, MF20 in green, S100b in red and synaptophysin in grey. Scale bars: 200 µm. Scale bar of zoomed picture (right): 50 µm. White dotted rectangle represents the zoom region represented on the right picture. White arrowhead indicates region where MN and MC are in close contact.

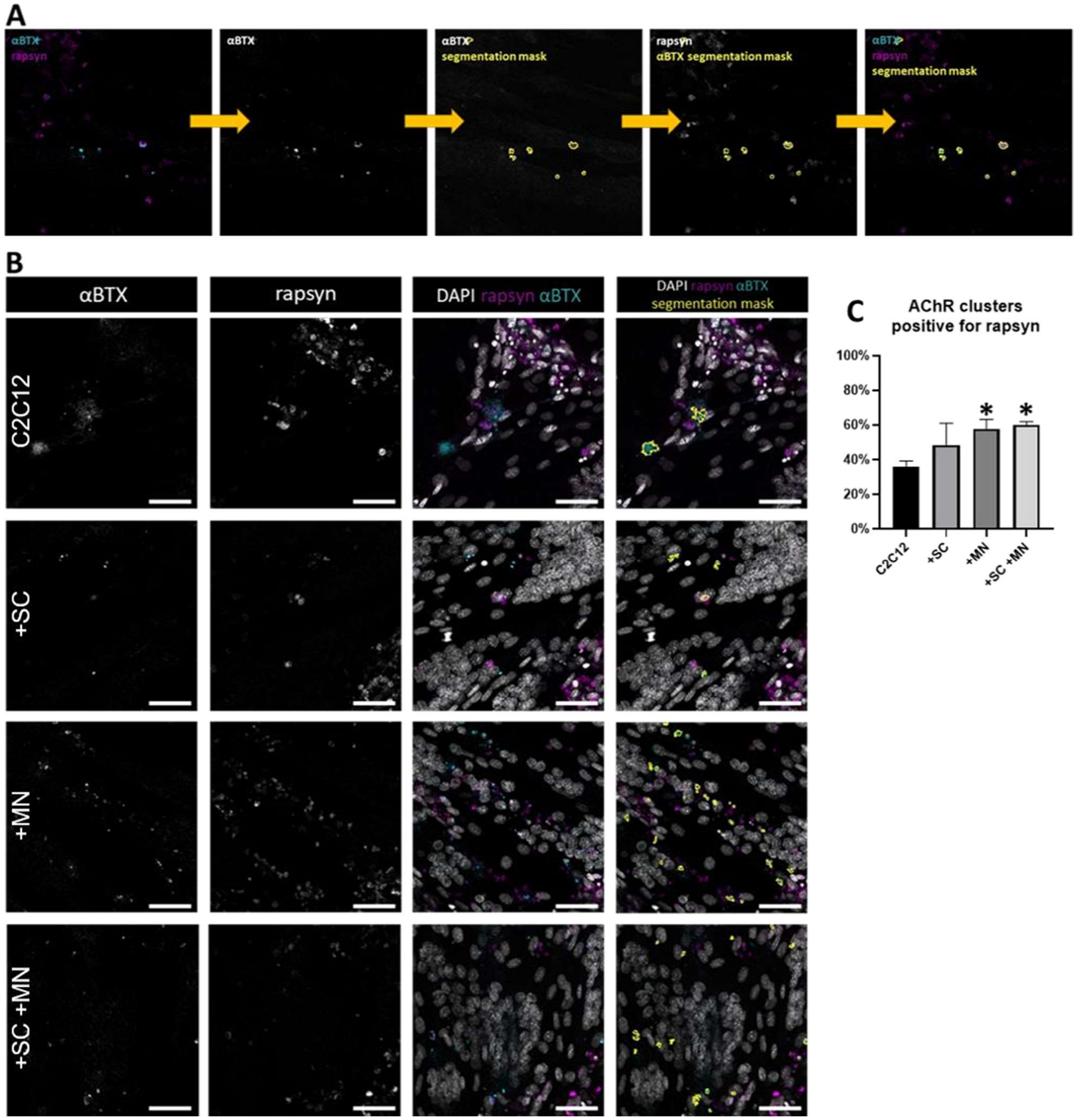

Supplementary Figure S6. Cocultures increase percentage of AChR clusters positive for rapsyn staining. (**A**) Cultures were stained for AChR using αBTX and co-stained for rapsyn. AChR clusters were segmented using the αBTX staining. αBTX segmentation ROIs were used to measure fluorescence intensity in the rapsyn channel. ROIs with a rapsyn staining intensity > (background mean intensity + 2x SD) were counted as positive. (**B**) Representative images of αBTX/rapsyn co-stainings and segmentation for all coculture conditions. Scale bars: 50 µm. (**C**) Percentage of AChR clusters positive for rapsyn in coculture conditions as indicated. Data presented as mean ± SD, n = 3. * $p < 0.05$.

Supplementary Table S1. Primary antibodies used for immunofluorescence stainings

Antibody target	Host species	Supplier	Cat. No.
**ChAT**	goat	Merck Millipore	AB144P
**Desmin**	rabbit	Abcam	ab32362
**Gap43**	mouse	Novus Bio	NBP-50052
**Hb9**	mouse	DSHB	81.5C10
**Isl1**	mouse	DSHB	40.2D6
**Ki67**	rabbit	Sigma Aldrich	AB9260
**Map2**	mouse	Synaptic Systems	188 011
**MF20**	mouse	DSHB	MF 20-c
**P0 (myelin protein zero)**	rabbit	Cell Signaling Technology	57518S
**Peripherin**	rabbit	Merck Millipore	AB1530
**Rapsyn**	rabbit	Santa Cruz Biotechnology	sc-28933
**S100b**	rabbit	Sigma Aldrich	HPA015768
**SOX10**	rabbit	Cell Signaling Technology	89356S
**SOX2**	rabbit	Synaptic Systems	347 003
**ßIII-tubulin**	guinea pig	Synaptic Systems	302 304
**Synaptophysin**	guinea pig	Synaptic Systems	101 011
**Tau**	guinea pig	Synaptic Systems	314 004
**vAChT**	rabbit	Synaptic Systems	139 103
**Vimentin**	mouse	Thermo Fisher Scientific	MA5-11883
